# Supplementary material for: Digital home monitoring for capturing daily fluctuation of symptoms; a longitudinal repeated measures study: Long Covid Multi-disciplinary Consortium to Optimise Treatments and Services across the NHS (a LOCOMOTION study)
Source: BMJ Open. 2023 Aug 8;13(8):e071428. doi: 10.1136/bmjopen-2022-071428 (PMC10414119; doi:10.1136/bmjopen-2022-071428)
Supplement: Supplementary data [file bmjopen-2022-071428supp001.pdf]

	**Constructs**	**Scale**	**Questions**	**Timing**
**1.Wellbeing**		NRS 11 point scale Sliding scale	1. **How well do you feel just now?**<br><br>Unwell – very well.	First survey of the day: suggest 9am but to be confirmed.<br>Delivered 6 times randomly stratified between 10am and 8pm.<br>Then at 9pm
**2.Sleep**	Quality<br>Amount /duration & how regular is sleep pattern?<br>Sleep latency (?)<br>Fractured sleep	4 point ordinal scale	1. **Thinking about your sleep last night, how would you rate the quality of your sleep?**<br><br>(Very good- very poor on sliding scale)<br>2. **What time did you go to bed last night?**<br>3. **What time did you get up this morning?**<br>4. **How long (in minutes) did it take you to fall asleep last night?**<br><br>(Options: 20 minutes or less, 20 to 60 minutes, 1-2 hours, more than 2 hours.)<br>5. **How many times did you wake up during the night?**<br><br>(Options: None, 1, 2-3, 4 or more)	First survey of the day: suggest 9am but to be confirmed
**3.Post exertion malaise**	PEM	1 hour or less, 1-3 hours, 4-8 hours, more than 8 hours.	1. **Thinking about today, did you experience worsening of**	Last survey of the day: suggest 9pm but to

			**your fatigue or Long Covid symptoms after minimal physical effort?** Yes/No<br><br>2. **Did you experience worsening of your fatigue or Long Covid symptoms after any mental effort?** Yes/No<br><br>3. **How long did this last?**<br><br>Options: 1 hour or less, 1-3 hours, 4-8 hours, more than 8 hours.	be confirmed.
**4.Activity**	Perceived effort Type – classified by mental/physical/social.	0 no effort -10 most effortful	1. **Thinking about what were you doing just before completing this survey, which one of the following best describes your activity:**<br><br>• Resting or sleeping<br>• Looking after myself (for example, getting dressed, eating)<br>• Mostly physical activity (for example walking, cleaning, exercising)	Delivered 6 times randomly stratified between 10am and 8pm.<br>Then at 9 am and 9 pm

			● Mostly mental activity (such as reading, using a computer or phone, working or studying)<br>● Socialising or talking with other people<br><br>**2. How effortful was that activity?**<br>Sliding scale: 0 no effort to 10 most effortful	
**5.Psychological factors**	Stress<br>Repetitive thinking	Not at all/a Little/Moderately, Very.	**1. Thinking about the last hour, to what extent have you?**<br><br>● Felt stressed<br>● Worried about your illness in the future<br>● Thought about your illness in the past.	Delivered 6 times randomly stratified between 10am and 8pm.<br>Then at 9 am and pm
**6.Impact on daily functioning**	Severity and frequency?	Yes/no	**1. Thinking about the last hour have you experienced any of the following**<br><br>● Problems with personal care (washing/dressing)<br>● Problems doing usual activities<br>● Feeling exhausted after an activity	Delivered 6 times randomly stratified between 10am and 8pm.<br>Then at 9 am and pm

			• Problems walking about<br>• Problems communicating with others<br>• Problems concentrating or remembering things	
**7.Symptoms**	Frequency and severity		1. At the moment are you experiencing:<br><br>**Breathlessness** Yes/ no<br>If yes, it branches to the question: please rate how severe your breathlessness is:<br>***1 = Mild problem;***<br>***2 = Moderate problem;***<br>***3 = Severe problem;***<br><br>2. **Fatigue** yes/no<br>Branch as above.<br><br>3. **Pain/discomfort** yes/no<br>Branch as above.<br><br>4. **Dizziness or palpitations** yes/no<br>Branch as above.<br><br>5. **Feeling anxious** yes/no<br>Branch as above.<br><br>6. **Feeling depressed** yes/no<br>Branch as above.	Delivered 6 times randomly stratified between 10am and 8pm.<br>Then at 9 am and pm

**7.Change**	Improvement or decline in health status	Modified Patients' Global Impression of Change (PGIC)	See full assessment	onto redcap questionnaire
**8.Participation and activity**	Participation	Oxford Participation and Activities Questionnaire (OxPAQ)	See full assessment	onto redcap questionnaire
**9.Symptom burden and functional impact**	Multi dimensional	C19YRS	See full assessment	onto redcap questionnaire

Appendix 1: Ecological Momentary Assessment Ecological Momentary Assessment EMA assessment
