# Supplementary material for: Digital home monitoring for capturing daily fluctuation of symptoms; a longitudinal repeated measures study: Long Covid Multi-disciplinary Consortium to Optimise Treatments and Services across the NHS (a LOCOMOTION study)
Source: BMJ Open. 2023 Aug 8;13(8):e071428. doi: 10.1136/bmjopen-2022-071428 (PMC10414119; doi:10.1136/bmjopen-2022-071428)
Supplement: Author's manuscript [file bmjopen-2022-071428.draft_revisions.pdf]

BMJ Open

BMJ Open is committed to open peer review. As part of this commitment we make the peer review history of every article we publish publicly available.

When an article is published we post the peer reviewers' comments and the authors' responses online. We also post the versions of the paper that were used during peer review. These are the versions that the peer review comments apply to.

The versions of the paper that follow are the versions that were submitted during the peer review process. They are not the versions of record or the final published versions. They should not be cited or distributed as the published version of this manuscript.

BMJ Open is an open access journal and the full, final, typeset and author-corrected version of record of the manuscript is available on our site with no access controls, subscription charges or pay-per-view fees (http://bmjopen.bmj.com).

If you have any questions on BMJ Open's open peer review process please email info.bmjopen@bmj.com

BMJ Open

Long Covid Multi-disciplinary consortium to Optimise Treatments and services across the NHS (LOCOMOTION): Symptoms and Activity Remote Monitoring protocol

Journal:	*BMJ Open*
Manuscript ID	bmjopen-2022-071428
Article Type:	Protocol
Date Submitted by the Author:	14-Mar-2023
Complete List of Authors:	Mansoubi, Maedeh; University of Exeter<br>Dawes, Joanna; University of Exeter<br>Bhatia, Aishwarya; University of Exeter<br>Vashisht, Himanshu; Dublin City University<br>Collett, Johnny; Oxford Brookes University, Department of Sport, Health and Social Work<br>Greenwood, Darren; University of Leeds, Biostatistics Unit<br>Ezekiel , Leisle ; University of Southampton<br>O'Connor, Daryl; University of Leeds,<br>Leveridge, Phaedra ; University of Exeter<br>Rayner, Clare; University of Leeds<br>Read, Flo; University of Exeter<br>Sivan, Manoj; University of Leeds,<br>Tuckerbell, Ian; University of Leeds<br>Ward, Tomas; Dublin City University<br>Delaney, Brendan; Imperial College London, Surgery and Cancer<br>Locomotion consortium, Locomotion consortium; University of Leeds<br>Dawes, Helen; University of Exeter, medical school
Keywords:	Rehabilitation medicine < INTERNAL MEDICINE, Fatigue, PAIN MANAGEMENT

SCHOLARONE™
Manuscripts

Authors:
Maedeh Mansoubi[1,2,3], Joanna Dawes[1], Aishwarya Bhatia[1], Himanshu Vashisht[4], Johnny Collett[5], Darren C. Greenwood[6], Leisle Ezekiel[7] , Daryl B. O'Connor[8], Phaedra Leveridge[1] Clare Rayner[9], Flo Read[1], Manoj Sivan[10], Ian Tucker-Bell[9], Tomas Ward[11], Brendan Delaney [12], **LOCOMOTION consortium,** Helen Dawes[1,2,3]

Collaborators:
LOCOMOTION consortium: Nawar Diar Bakerly, Mauricio Barahona, Alexander Casson, Jonathan Clarke, Vasa Curcin, Helen Davies, Carlos Echevarria, Sarah Elkin, Rachael Evans, Zaccheus Falope, Ben Glampson, Trisha Greenhalgh, Stephen Halpin, Mike Horton, Joseph Kwon, Simon de Lusignan, Gayathri Delanerolle, Erik Mayer, Harsha Master, Ruairidh Milne, Ghazala Mir, Jacqui Morris, Amy Parkin, Stavros Petrou, Anton Pick, Nick Preston, Amy Rebane, Emma Tucker, Ana Belen Espinosa Gonzalez, Sareeta Baley, Annette Rolls, Emily Bullock, Megan Ball, Shehnaz Bashir, Joanne Elwin, Denys Prociuk, Iram Qureshi, Samantha Jones

Affiliation:

1. Medical School, University of Exeter, Exeter, UK
2. Biomedical Research Center, Medical School, Faculty of Health and Life sciences, University of Exeter, Exeter, EX1 2LU United Kingdom
3. Intersect@Exeter, Medical School, University of Exeter, Exeter, UK
4. The Wild Research Limited, Dublin, Ireland
5. Department of Sport, Health and Social Work, Oxford Brookes University
6. Academic Department of Rehabilitation Medicine, University of Leeds, Leeds, UK
7. Southampton University
8. School of Psychology, University of Leeds, UK
9. Patient Advisory Group (PAG) Representative
10. Faculty of Medicine and Health, School of Medicine, University of Leeds, UK
11. Insight SFI Research Centre for Data Analytics, Dublin City University, Dublin, Ireland
12. Faculty of Medicine, Department of Surgery & Cancer, Imperial College London

Corresponding Author
Helen Dawes
h.dawes@exeter.ac.uk
Medical school, University of Exeter
www.exeter.ac.uk
Medical School Building, College of Medicine and Health, St Lukes Campus, Heavitree Road, Exeter, EX12LU

Word count: 4289

For peer review only

Long Covid Multi-disciplinary consortium to Optimise Treatments and services across the NHS (LOCOMOTION): Symptoms and Activity Remote Monitoring protocol

Abstract

Introduction:

A substantial proportion of COVID-19 survivors continue to have symptoms more than three months after infection, especially of those who required medical intervention. Lasting symptoms are wide-ranging, and presentation varies between individuals and fluctuates within an individual. Improved understanding of undulation in symptoms and triggers may improve efficacy of healthcare providers and enable individuals to better self-manage their Long Covid. We present a protocol where we aim to develop and examine the feasibility and usability of digital home-monitoring for capturing daily fluctuation of symptoms in individuals with Long Covid and provide data to facilitate a personalised approach to the classification and management of Long Covid symptoms.

Methods and Analysis:

This study is a longitudinal prospective cohort study of adults with Long Covid accessing 10 National Health Service (NHS) rehabilitation services in the UK. We aim to recruit 400 people from participating NHS sites. At referral to study, six weeks and 12 weeks, participants will complete demographic data (referral to study) and clinical outcome measures, including Ecological Momentary Assessment (EMA) using personal mobile devices. EMA items are adapted from the COVID-19 Yorkshire Rehabilitation Scale (C19-YRS) items and include self-reported activities, symptoms, and psychological factors. Passive activity data will be collected through wrist-worn sensors. We will use latent class growth models to identify trajectories of experience, potential phenotypes defined by co-occurrence of symptoms, and interrelationships between stressors, symptoms, and participation in daily activities. We anticipate that n=300 participants provide 80% power to detect a 20% improvement in fatigue over 12 weeks in one class of patients relative to another.

Ethics and Dissemination:

The study was approved by the Yorkshire & The Humber—Bradford Leeds Research Ethics Committee (ref: 21/YH/0276). Findings will be disseminated in peer-reviewed publications and presented at conferences.

Trial registration number: ISRCTN15022307
Keywords: Long covid, Home monitoring, Ecological Momentary Assessment

For peer review only

Strengths and Limitations

- Recruitment of participants across multiple NHS Long Covid clinics across England.
- Readily available technology is used to monitor symptoms in real-time in the context of daily life.
- The reliability and validity of EMA constructs are limited.
- EMA completion rates and participant assessment reactivity is limited.
- The longitudinal and remote monitoring aspect of the study means there is a risk of some participants not engaging with the study and participant attrition.

For peer review only

INTRODUCTION

Since the start of the COVID-19 pandemic in 2019, over 480 million people across the globe have had confirmed COVID-19 infection (1). A substantial proportion of COVID-19 survivors will continue to have symptoms more than three months after infection, especially, but not restricted to, those requiring medical intervention for the infection (2–5). The term Long COVID is used to define the long-lasting symptoms of COVID-19 and includes ongoing symptomatic COVID-19 (from 4 to 12 weeks) and post-COVID-19 syndrome (12 weeks or more) (6). In the UK alone, an estimated 1.5 million (5) and over 100 million people globally (2) are living with self-reported Long Covid (5).

The presentation of Long Covid is variable with a wide range of symptoms which most commonly include general weakness, malaise, fatigue, breathlessness, and impaired concentration (7–9). Long Covid has been described as having "undulating symptoms" where individuals may experience feeling well but then have a re-occurrence of symptoms, sometimes in response to triggers such as stress or physical activity (10). The need for a greater degree of definitional stringency and clearer classification of symptom incidences, number, severity, trajectory and duration for clinical trials has been highlighted (11). To address this, and a need to improve access to multi-disciplinary health care for the large numbers of people reporting Long Covid (10), we propose exploring the use of remote monitoring methods with readily available technology as a potential tool to assist patients and healthcare professionals in the clearer classification of symptoms and a better understanding of symptom management and help with recovery (12,13).[1]

There are currently no validated monitoring systems to classify and regularly check a patient's symptoms and report them to doctors and other healthcare professionals. A recent systematic review including 272 articles demonstrated that remote patient monitoring methods could improve patient support and the efficiency of the therapy (14). According to this review, smartphone applications and wireless devices are the most common Remote Patient Monitoring (RPM) methods (14). Given the lack of evidence on a valid and feasible method to support patients and health systems with COVID symptom RPM and management, this work package of the LOCOMOTION study (15) aims to develop and examine the feasibility and usability of home monitoring (using smartphone applications and wearable sensors) for capturing the daily fluctuation of symptoms in

[1]Abbreviations:

Long Covid Multi-disciplinary consortium to Optimise Treatments and services across the NHS (LOCOMOTION), COVID-19 Yorkshire Rehabilitation Scale (C19-YRS), Remote Patient Monitoring (RPM), Post-exertional malaise (PEM), University of California, San Diego Brief Assessment of Capacity to Consent Brief Assessment of Capacity to Consent (UBACC), Research Electronic Data Capture (REDCap), Vector Autoregressive modelling (VAR), Markov chain Monte Carlo (MCMC), Ecological Momentary Assessment (EMA).

For peer review only - http://bmjopen.bmj.com/site/about/guidelines.xhtml

real-time, recognising adverse triggers across patients from a range of demographic groups and aid self-management.

The primary objectives of the study are:

1. To quantify the extent to which total and specific (cognitive, physical, and social) activities, sleep and rest predict subsequent incidence, severity, number and duration of Long Covid symptoms, including post-exertional malaise (PEM).
2. To identify different groups or classes of people who respond to physical or mental effort differently in regards to PEM, other symptom incidences, duration, number and severity.

The secondary objectives of the study are to:

3. Quantify the extent to which the prevalence or severity of specific symptoms predicts subsequent activity.
4. Quantify the relationship between sleep duration and quality with Long Covid symptoms.
5. Explore the relationship between different component contributions to the overall level of physical activity (frequency, intensity, recovery, duration) and subsequent symptoms.
6. Explore the associations between different timing or degree of exposure to (cognitive, physical and social) activities in terms of cumulative exposure (e.g. multiple triggers in a short space of time or repeated triggers over the day), along with the time-lagged onset of symptoms or exacerbation of symptom severity.
7. Determine the extent of the relationship between physiological variables and activity and symptom severity for different groups of activities and symptoms. (Substudy 1).
8. Describe the stability of the activity, symptoms and the interrelationship in people with Long Covid.
9. Describe the variability in activities, dose of activities and symptom severity
10. Explore the moderating effects of living with physical health and/or mental health conditions (including depression and anxiety) on the relationships between the triggers and symptoms of Long Covid (as outlined in objectives 1 and 2).
11. Describe the concurrent validity of components of the home monitoring system (Substudy 1)
12. Investigate usability and acceptability of home monitoring system and explore patient experiences of Long Covid (Substudy 2)

METHODS AND ANALYSIS

This is a workstream of the LOCOMOTION health service study (trial registration number ISRCTN15022307) (15) . The study follows Strobe guidelines https://www.equator-network.org/reporting-guidelines/strobe/.

For peer review only

Design and setting: Using a longitudinal study design with repeated measures and a nested substudy we aim to recruit a purposive sample of patients with (self-reported/GP diagnosed) Long Covid who have been referred to one of the ten participating NHS rehabilitation clinics (see figure 1). We will recruit 400 participants with the anticipation of a 30% dropout, giving 300 patients (40 per site) using purposive sampling.

At referral to study, at six weeks, and 12 weeks, participants will complete demographic data (on referral to study) and clinical outcome measures, including Ecological Momentary Assessment (EMA) and passive activity data over seven days using personal mobile devices. EMA symptoms items are adapted from the COVID-19 Yorkshire Rehabilitation Scale (C19-YRS) items and include self-reported activities, symptoms and psychological factors. Passive activity data will be collected through wrist-worn sensors. EMA consists of sampling an individual's experiences and subjective states as they occur in real-time in the individual's natural environment (16). There will be two embedded sub-studies. Sub study 1 will include additional home digital physiological measures of heart rate variability (HRV) to test biofeedback (improving symptoms by increasing HRV). Sub study 2 will use qualitative interviews to assess the usability and acceptability of using home monitoring (via smartphones) of triggers and symptoms of Long Covid at clinic appointments and explore patient experiences of Long Covid.

Inclusion criteria

We will include all adults who are 18 years and over, able to participate in the study (i.e., able to complete the EMA and use the wearable technology), able to give informed consent and have English language proficiency to the level of conducting health consultations. Participants will not be included or excluded based on past or current illness or medication at the time of the presumed Covid infection; however, they will be asked to describe these at the start of this study.

Exclusion criteria

Participants will be excluded who are under 18 years old, unable to use the EMA app technology or use the wearable technology, unable to understand the language used in the EMA or are known to be pregnant. We will also exclude those with a known previous diagnosis of dementia or cognitive impairment that would prevent ability to participate in the use of the EMA or wearable technologies.

Main study

With broad assumptions, based on pilot data from our rehabilitation clinics, we anticipate n=300 participants would provide approximately 80% power to detect 20% improvement in fatigue over 12 weeks in one class of patients relative to another, e.g., a stable symptom group with fatigue scores

For peer review only

of 5 at each time point, compared to a group gradually improving from 5 to 4, with within-person correlation 0.7, residual variance 2.0, and 30% with the symptom pattern. We anticipate up to 30% dropout across follow-up, so we initially aim to recruit up to n=400.

Sub study 1: (Reliability and physiological phenotyping study of home monitoring system)

This study will invite 50 participants, allowing for dropouts and leaving a sample size of approximately 30 participants to ensure meeting central limit theorem criteria for reliability analysis and between 25-50 for meeting feasibility criteria (see (17))

Sub study 2: (Usability and acceptability of home monitoring system)

This study will invite 20 patients who have taken part in the main monitoring study to be interviewed and 10 clinicians working in one of the collaborating Long Covid clinics. This sample size should ensure sufficient 'information power' to address the research questions (18).

Recruitment

Each of the ten participating Long Covid clinics has a clinical research lead who is responsible for inviting patients to participate in the study on referral. Potential participants will be notified of the opportunity to take part in the study by the clinical research lead at each site and provided with a link to an online form to register their expression of interest and check that they meet the study inclusion criteria. Intake will be capped when recruitment has achieved the study sample size, and further applicants will receive an email response that the study is closed but that they will be contacted again when it is open.

Data collection

Baseline and follow-up data collection

Baseline and follow-up measures will be collected using the online data capture platform REDCap. On entry to the study, participants will also complete the C19-YRS and provide information about their demographics (age, height, weight, sex, ethnicity, postcode, date of birth, marital status, housing, education, employment, smoking, disability, past medical history, drug history, covid history and care). Additionally, at weeks one, six and twelve, participants will complete the COVID-19 Yorkshire Rehabilitation Scale (C19-YRS) (19) and provide information about their physical activity levels, fatigue, quality of life and participation, wellbeing, anxiety and rumination, worry, depression and post-exertional malaise.

Wearables for home monitoring of fluctuation of symptoms

For peer review only

At referral to the study, at six weeks and 12 weeks, participants will be asked to perform ecological momentary assessments (EMA) over 7 days using personal mobile devices and wearable sensors and accelerometers. The EMA is delivered using the AthenaCX platform, a secure and GDPR-compliant research survey platform (20) which works on Apple and android phones. The EMA includes five time-contingent surveys (sent between 8.45 am and 9.15 pm) of self-reported activities, symptoms and psychological factors (see Appendix 1). EMA questions were adapted from the C19-YRS items to enable home monitoring of symptom fluctuation and trigger identification, and from items developed for the UK COVID-19 Mental Health and Wellbeing study (21). Pilot work has demonstrated that this approach is feasible and acceptable to Long Covid patients.

Accelerometer sensor

The daily amount of physical activity and sedentary time will be objectively assessed using the Axivity AX3, a “wrist-worn” tri-axial accelerometer designed by Open Lab, Newcastle University, UK. AX3 data will be downloaded using OMGUI software (open movement [V.1.0.0.37]). The Axivity AX3 is a valid and reliable method for the measurement of physical activity and sedentary behaviour (22)

Sub study 1 (Reliability and physiological phenotyping study of home monitoring system):

Reliability and physiological phenotyping study of 50 patients in Oxford to include additional home digital physiological measures HRV (heart rate variability)

> Method: Fifty patients from the clinic in Oxford will be invited to participate in the following voluntarily and will be able to opt-in or out of any of the components at each testing time point: digitally and remotely monitoring physiological measures via a wrist-worn or a chest strap heart rate sensor, based on participants’ preference and capability to measure heart rate and heart rate variability. This aims to explore the potential to use measuring heart rate and heart rate variability as feedback for clinical purposes.

Sub-study 2 process evaluation (Usability and acceptability of home monitoring system):

Usability, acceptability and co-design qualitative study with 20 patients and ten clinicians and a clinician survey to assess the usability and acceptability of using home monitoring (via smartphones) of triggers and symptoms of Long Covid at clinic appointments and explore patient experiences of Long Covid.

> Method: Sub-study 2 will be a co-design study between 20 patients and ten clinicians. It will use a survey to explore symptom behaviour and establish the utility of prospective symptom fluctuation and triggers for self-management. We will conduct a short debrief interview with up to 20 participants during either week three or week four of their study.

For peer review only

This will be conducted by phone/video call and recorded. The interview will be structured around the usability dimensions of efficiency, effectiveness, and satisfaction. In particular, the interview will ask about both the apps and using them in daily life, any issues identified and any suggestions for improvement to the app or the study design. The interview will include a "would you recommend to someone you know?" question and explore reasons for this. The interview will also include a think-aloud component in which the interviewer asks the participant to go through a data entry cycle and describe what they are thinking or doing as they go along. Towards the end of the interview, the interviewee will be asked to complete the 10-item System Usability Scale. Participants will also be interviewed about the experiences of Long Covid.

For clinicians, the acceptability and usability assessment will focus on content, design, language and adaptability of daily monitoring information in the clinic environment and explore views on the usefulness of this approach to support Long Covid patients. Semi-structured interviews will be conducted either face to face or via the secure web video conferencing system.

Data cleaning

Reported values more than 3 standard deviations from the mean will be double-checked with original data sources. Observations with Studentised residuals greater than 3, Cook's distance greater than 1, and leverage of $h>0.3$ will be used to detect influential data points in subsequent models. No data will be excluded without evidence of error or implausibility, other than through sensitivity analyses.

Descriptive statistics

Patient characteristics (demographics, clinical history, exposures and outcomes) will be tabulated overall, by sex, by 2 to 5 broad age bands derived from the data to provide similar numbers in each age group, and by 2 to 5 fatigue bands, similarly derived, with sensible rounding of thresholds (e.g. to nearest 5 years or to nearest 1 point on scale).

Visual analogue scales and measures will be presented as median and interquartile range or mean and standard deviation, dependent on the underlying distributions of the scores.

The characteristics of consenting participants will be compared with the characteristics of all clinic patients, to investigate whether the participating patients differ systematically from

For peer review only

those not participating. Characteristics of participants with incomplete data will be compared to those with complete data.

Statistical modelling

Primary objective 1:

Multilevel multivariate vector autoregressive (VAR) modelling will be used to investigate the associations between C19-YRS symptom scores, and the activities reported in the EMA, taking account of the hierarchical/nested multilevel structure of the data (Objective 1). We will construct multilevel multivariate vector autoregressive models within the Dynamic Structural Equation Model (DSEM) module in Mplus 8.6 or WinBUGS 1.4 to partition total variability across individuals and time points into within- and between-person components. We anticipate using non-informative priors and two independent Markov chain Monte Carlo (MCMC) chains, allowed to burn in until distributions are stable.

The within-person component will be modelled using a vector autoregressive (VAR) time-series model of order 1. This model will be used to predict the outcomes at one-time point using all predictor variables at the previous time point. This will allow estimation of the within-person associations between symptom outcomes and potential triggers, the autoregressive effects that the severity of the outcome variable has on itself at the next time point, and the cross-lagged effects of one variable on another variable at the next time point. Both autoregressive and cross-lagged parameters will be modelled as random effects that vary across individuals. We anticipate fitting both random intercepts and slopes but assuming these are independent of each other. Robustness to this assumption will be assessed in sensitivity analyses, where sufficient degrees of freedom and complexity of models allow.

We will estimate the association of between-individual differences in the random-effects with variation in other variables measured at the between-person level, e.g. whether change symptoms over time are associated with within-person dynamics between symptom severity and potential triggers such as activities and effort, by specifying the change in symptom severity as the outcome variable and the random effects of the cross-lagged and autoregressive parameters as predictors. All variables and parameters will be centred, standardised or detrended where appropriate to facilitate correct analysis and interpretation.

To allow for both shorter and longer-term lag effects between triggers and outcomes, analyses will be conducted first with each EMA as the time point (e.g. to compare mental

For peer review only

effort at one assessment with fatigue at the next), then repeated using the whole day as the timepoint, aggregating over the 6 EMAs that day (e.g. to compare impact of physical effort one day on symptoms the next day). This will also be used for any outcomes only measured once in a day.

The same methods will be applied to secondary objective 3 (symptoms predicting subsequent activity), objective 4 (timing, duration and quality of sleep), objective 5 (components of physical activity), objective 6 (timing and cumulation of activities), and in part to objectives 7 (heart rate variability and physiological phenotyping) and 8 (stability of association).

Primary objective 2:

Latent class growth models will be used to identify different types of symptom trajectories experienced by those living with Long Covid, potential phenotypes defined by co-occurrence of symptoms, and any interrelationships between triggers, symptoms, and participation in daily activities, personal care such as washing or dressing, usual activities, problems walking about, problems communicating with others, problems with concentration or remembering things (Objective 2). The extent of these between-person and within-person day-to-day fluctuations will be quantified, and the potential for characteristics to predict subsequent improvement over time. The number of classes will be based on information criteria (e.g. BIC and AIC) and clarity of interpretation of the domains identified.

Functional data analysis will also be used, where appropriate, to characterise features of long-term symptom trajectories, such as variation and speed of change. The extent of these between-person and within-person day-to-day fluctuations will be quantified, and the potential for characteristics to predict subsequent improvement over time.

These methods will also be applied in part to objectives 7 (heart rate variability and physiological phenotyping) and 8 (stability of association).

All statistical analyses will be conducted using SPSS 28, R, Stata 17 and MPlus 8.6 or WinBUGS 1.4.

Bias

Potential confounding

Potential confounders will be identified based on directed acyclic graphs, but as the aim is prediction, we will also include potential competing exposures in models.

Missing and incomplete data

For peer review only

The latent class growth models utilise mixed-effects modelling that allows for partially incomplete repeated measures across participants.

Exclusions, sensitivity analyses and subgroup analyses

Sensitivity analyses will be conducted to assess robustness to different types of trajectories for men vs women, hospitalised vs non-hospitalised, individuals with existing mental health conditions vs individuals without existing mental health conditions, different waves of acute infection as a proxy for variant, broad age band.

We have previously described sensitivity analysis assessing robustness of the models to the timeframe used for the VAR analysis, i.e. EMA or summary measure over the whole day   We will also assess robustness of the model to 2nd order time-lags, in addition to the 1st order time-lags, where the data allows.

We assume independence of random intercepts and slopes but will assess robustness to this correlation structure where the data allows.

As sensitivity analysis to the effects of incomplete data, we will repeat excluding any days with fewer than 2 EMAs.

Thematic analysis

Thematic content analysis of interview transcripts will be used to investigate the usability and perceived usefulness of the digital measurement tools (by clinicians and patients) to support self-management and rehabilitation. It will be also used to explore participants' experiences of Long Covid triggers and symptoms. All qualitative data collection for the process evaluation will be carried out by a research team member with qualitative research experience. Interviews will be transcribed verbatim and analysed using thematic analysis methods which involves familiarisation with the interview, coding, developing and applying an analytical frame-work, charting data into the analytical framework for analysis (23).   Interview transcripts will be coded using NVivo software (or similar software). Each interview will be independently coded by two reviewers. After coding four transcripts, reviewers will compare codes and discrepancies will be discussed and resolved prior to coding the remaining transcripts. The interim analysis will be conducted following an initial sample of 150 patients to determine whether saturation of themes had been reached. Mean (±standard deviation) or median and interquartile range values will be used as appropriate to summarise participants' demographic data.

For peer review only

Interviews will be analysed thematically initially to explore usability factors. Due to the focus of this process evaluation, the personal documentation will be initially subjected to content analysis—with a more detailed thematic analysis applied if required. Data will be reviewed in light of any contradictions and will guide a member-checking process with participants, which will be conducted prior to the write-up and dissemination of findings. Findings will be separated into the process and measure ready for reporting, but with consideration being given to where/if the doing of any process is a major contributor to the outcomes or perceived experience of participants. At this point, we will explore possible mechanisms for any observed effects, to generate change objectives, and behavioural outcomes.

Public patient involvement and engagement

The team have regular public patient involvement (PPI) meetings, and the study was designed in consultation with Long Covid patients on how COVID-19 has disproportionately affected different communities. The LOCOMOTION study has a seven-member core PPI Advisory Group (PAG) that includes different cultural, ethnic, and socioeconomic groups. They have contributed to the proposal research planning meetings and met separately to examine and develop the research aim, objectives, and questions ensuring these align with the key research priorities of patients with Long Covid and represent different patient needs.

Ethics and Dissemination

The study was approved by the Yorkshire & The Humber—Bradford Leeds Research Ethics Committee (ref: 21/YH/0276) and conforms with the Declaration of Helsinki.

Participants consent to participate following an e-consent process. The e-consent form will be implemented through a secure web-based online survey accessible by computer, mobile phone, or tablet. The e-consent process also includes questions about the person's understanding of the study based on the Brief Assessment of Capacity to Consent (UBACC) (24); participants need to answer at least 6 of 8 questions correctly to be eligible for the study. This process queries the person's understanding of the consent form and has been used in over 200 research studies worldwide. A parallel consent process using telephones is available for those participants who are not able to use, or do not have access to the internet. Potential participants will be telephoned when the clinical research lead indicates this is the preferred mode of contact and communication to the research team. A call will be set up for one of the research teams to provide them with information. If the potential participant is interested, a second call will elicit their consent over the phone using the

For peer review only

same script as e-consent and conduct the screening process using the same questionnaire. The questions on the capacity to consent quiz will be asked orally.

Findings will be disseminated through manuscript publications in peer-reviewed journals and conference presentations.

DISCUSSION

The overarching aim of this study is to optimise the classification and self-management of Long Covid by tracking symptoms in real-time in the context of daily life, and by using a data-driven approach to identify adverse triggers of symptoms and phenotypes of Long Covid. These findings also aim to extend knowledge of the trajectory of Long Covid, particularly interactions between Long Covid symptoms and activity-related behaviours. Understanding the relationship between Long Covid symptoms and everyday activity is essential for supporting effective self-management of Long Covid, particularly on achieving balance of activity, priority of activity in order to manage social, family and work commitments. The study findings will help facilitate a personalised approach to the management of Long Covid based on ecologically valid self-report data.

Additionally, we will evaluate the usability of digital home monitoring by patients and clinicians. Taken together the findings of these studies will help support the potential development of a digital home monitoring, self-management platform for use with Long Covid patients. It is hoped that a future home monitoring platform will enable the provision of cost and time-effective care.

Strengths and limitations

Strengths

Key strengths of this study are the planned recruitment of participants through multiple NHS Long Covid clinics across England, and the use of readily available technology and real-time monitoring of symptoms in the context of daily life. These aspects of the study design increase the generalizability and ecological validity of the study findings.

Limitations

There are several known methodological limitations to ecological momentary assessment: the reliability and validity of EMA constructs, EMA completion rates and participant assessment reactivity (25). In addition, the longitudinal and remote monitoring aspect of the study means there is a risk of some participants not engaging with the study, and of participant attrition. We will use several strategies to minimise these limitations. Firstly, we optimised the face and construct validity of the EMA questions by using single constructs items from the C19-YRS (19) and PEM (26). In addition, the EMA was tested by people with Long Covid and clinicians with expertise in Long Covid

For peer review only

to check the clarity and relevance of questions. We also tested the EMA schedule with people with Long Covid to ensure the schedule was acceptable and to optimise completion rates. We planned bursts of assessment (7 days repeated three times) rather than extended periods, thereby reducing the burden on the participant. Finally, we recognise the need to support participants through the first stages of using the EMA app; therefore, each participant will have an initial phone call from a member of the research team and subsequently have ongoing contact with the team via email or telephone.

Author contributions

All the below contributed to study design, drafting editing or revising paper, and have given final approval and agreed to accountability.

Maedeh Mansoubi, Joanna Dawes, Johnny Collett, Leisle Ezekiel, and Helen Dawes, Brendan Delaney, Darren C. Greenwood[6], Daryl B. O'Connor, Clare Rayner, Manoj Sivan, Ian Tucker-Bell, Tomas Ward, Himanshu Vashisht, Aishwarya Bhatia, Flo Read and Phaedra Leveridge

LOCOMOTION consortium authors contributed in the protocol design.

Funding statement: This project has been funded by NIHR, grant reference number which is: COV-LT2-0016, Trial registration number NCT05057260, ISRCTN15022307. This work was supported by NIHR grant reference number [COV-LT2-0016].

Acknowledgement: This study was funded/supported* by the National Institute for Health and Care Research Exeter Biomedical Research Centre. Professor Helen Dawes and Dr Maedeh Mansoubi's work is supported by the National Institute for Health and Care Research Exeter Biomedical Research Centre. Dr Johnny Collett is funded by the NIHR Oxford Health Biomedical Research Centre. The views expressed are those of the author(s) and not necessarily those of the NIHR or the Department of Health and Social Care.

Competing interests statement: All authors declare that they have no conflicts of interest.

For peer review only

REFERENCES

1. WHO. WHO Coronavirus (COVID-19) Dashboard [Internet]. 2022 [cited 2022 Sep 23]. Available from: https://covid19.who.int/

2. Chen C, Haupert SR, Zimmermann L, Shi X, Fritsche LG, Mukherjee B. Global Prevalence of Post-Acute Sequelae of COVID-19 (PASC) or Long COVID: A Meta-Analysis and Systematic Review. J Infect Dis. 2022;19. DOI: 10.1093/infdis/jiac136

3. Fernández-de-las-Peñas C, Palacios-Ceña D, Gómez-Mayordomo V, Florencio LL, Cuadrado ML, Plaza-Manzano G, et al. Prevalence of post-COVID-19 symptoms in hospitalized and non-hospitalized COVID-19 survivors: A systematic review and meta-analysis. Eur J Intern Med. 2021;92(May):55–70. DOI: 10.1016/j.ejim.2021.06.009

4. Sugiyama A, Miwata K, Kitahara Y, Okimoto M, Abe K, E B, et al. Long COVID occurrence in COVID-19 survivors. Sci Rep. 2022;12(1):1–11. DOI: 10.1038/s41598-022-10051-z

5. ONS. Prevalence of ongoing symptoms following coronavirus (COVID-19) infection in the UK: 1 September 2022 [Internet]. 2022 [cited 2022 Sep 1]. Available from: https://www.ons.gov.uk/peoplepopulationandcommunity/healthandsocialcare/conditionsanddiseases/bulletins/prevalenceofongoingsymptomsfollowingcoronaviruscovid19infectionintheuk/1september2022

6. NICE. COVID-19 rapid guideline: managing the long-term effects of COVID-19 [Internet]. 2021 [cited 2022 Mar 29]. Available from: https://www.nice.org.uk/guidance/ng188

7. Aiyegbusi OL, Hughes SE, Turner G, Rivera SC, McMullan C, Chandan JS, et al. Symptoms, complications and management of long COVID: a review. J R Soc Med. 2021;114(9):428–42. DOI: 10.1177/01410768211032850

8. Raveendran A V., Jayadevan R, Sashidharan S. Long COVID: An overview. Diabetes Metab Syndr Clin Res Rev. 2021;15(3):869–75. DOI: 10.1016/j.dsx.2021.04.007

9. Michelen M, Manoharan L, Elkheir N, Cheng V, Dagens A, Hastie C, et al. Characterising long COVID: A living systematic review. BMJ Glob Health. 2021;6(9):1–12. DOI: 10.1136/bmjgh-2021-005427

10. NIHR. Living with Covid19 – Second review [Internet]. 2021 [cited 2021 Mar 1]. Available from: https://evidence.nihr.ac.uk/themedreview/living-with-covid19-second-review/

11. Ceban F, Leber A, Jawad MY, Yu M, Lui LMW, Subramaniapillai M, et al. Registered clinical trials investigating treatment of long COVID: a scoping review and recommendations for research. Infect Dis. 2022;54(7):467–77. DOI: 10.1080/23744235.2022.2043560

12. O'Connor DB, Aggleton JP, Chakrabarti B, Cooper CL, Creswell C, Dunsmuir S, et al. Research priorities for the COVID-19 pandemic and beyond: A call to action for psychological science. Br J Psychol. 2020;111(4):603–29. DOI: 10.1111/bjop.12468

13. Chiauzzi E, Rodarte C, DasMahapatra P. Patient-centered activity monitoring in the self-management of chronic health conditions. BMC Med. 2015;13(1):1–6. DOI: 10.1186/s12916-015-0319-2

For peer review only

14. de Farias FAC, Dagostini CM, Bicca Y de A, Falavigna VF, Falavigna A. Remote Patient Monitoring: A Systematic Review. Telemed E-Health. 2020;26(5). DOI: 10.1089/tmj.2019.0066

15. Sivan M, Greenhalgh T, Darbyshire JL, Mir G, O'Connor RJ, Dawes H, et al. LOng COvid Multidisciplinary consortium Optimising Treatments and servIces acrOss the NHS (LOCOMOTION): protocol for a mixed-methods study in the UK. BMJ Open. 2022;12(5):e063505. DOI: 10.1136/bmjopen-2022-063505

16. Shiffman S, Stone AA, Hufford MR. Ecological momentary assessment. Annu Rev Clin Psychol. 2008;4:1–32. DOI: 10.1146/annurev.clinpsy.3.022806.091415

17. NIHR. Justify sample size for a feasibility study [Internet]. [cited 2022 Mar 1]. Available from: https://www.rds-london.nihr.ac.uk/resources/justify-sample-size-for-a-feasibility-study/

18. Malterud K, Siersma VD, Guassora AD. Sample Size in Qualitative Interview Studies: Guided by Information Power. Qual Health Res. 2016;26(13):1753–60. DOI: 10.1177/1049732315617444

19. O'Connor RJ, Preston N, Parkin A, Makower S, Ross D, Gee J, et al. The COVID-19 Yorkshire Rehabilitation Scale (C19-YRS): Application and psychometric analysis in a post-COVID-19 syndrome cohort. J Med Virol. 2022;94(3):1027–34. DOI: 10.1002/jmv.27415

20. AthenaCX. AthenaCX [Internet]. [cited 2022 Nov 6]. Available from: https://www.athenacx.com/

21. O'Connor DB, Wilding S, Ferguson E, Cleare S, Wetherall K, McClelland H, et al. Effects of COVID-19-related worry and rumination on mental health and loneliness during the pandemic: longitudinal analyses of adults in the UK COVID-19 mental health & wellbeing study. J Ment Health. 2022;0(0):1–12. DOI: 10.1080/09638237.2022.2069716

22. De Craemer M, Verbestel V. Comparison of outcomes derived from the actigraph GT3X+ and the axivity AX3 accelerometer to objectively measure 24-hour movement behaviors in adults: A cross-sectional study. Int J Environ Res Public Health. 2022;19(1):4–11. DOI: 10.3390/ijerph19010271

23. Braun V, Clarke V. Using thematic analysis in psychology. Qual Res Psychol. 2006;3(2):77–101.

24. NICE. Decision-making and mental capacity [Internet]. 2018 [cited 2022 Apr 23]. Available from: https://www.nice.org.uk/guidance/ng108

25. Ram N, Brinberg M, Pincus AL, Conroy DE. The Questionable Ecological Validity of Ecological Momentary Assessment: Considerations for Design and Analysis. Res Hum Dev. 2017;14(3):253–70. doi: 10.1080/15427609.2017.1340052

26. Cotler J, Holtzman C, Dudun C, Jason L. A Brief Questionnaire to Assess Post-Exertional Malaise. Diagnostics. 2018;8(3):66. doi: 10.3390/diagnostics8030066

For peer review only

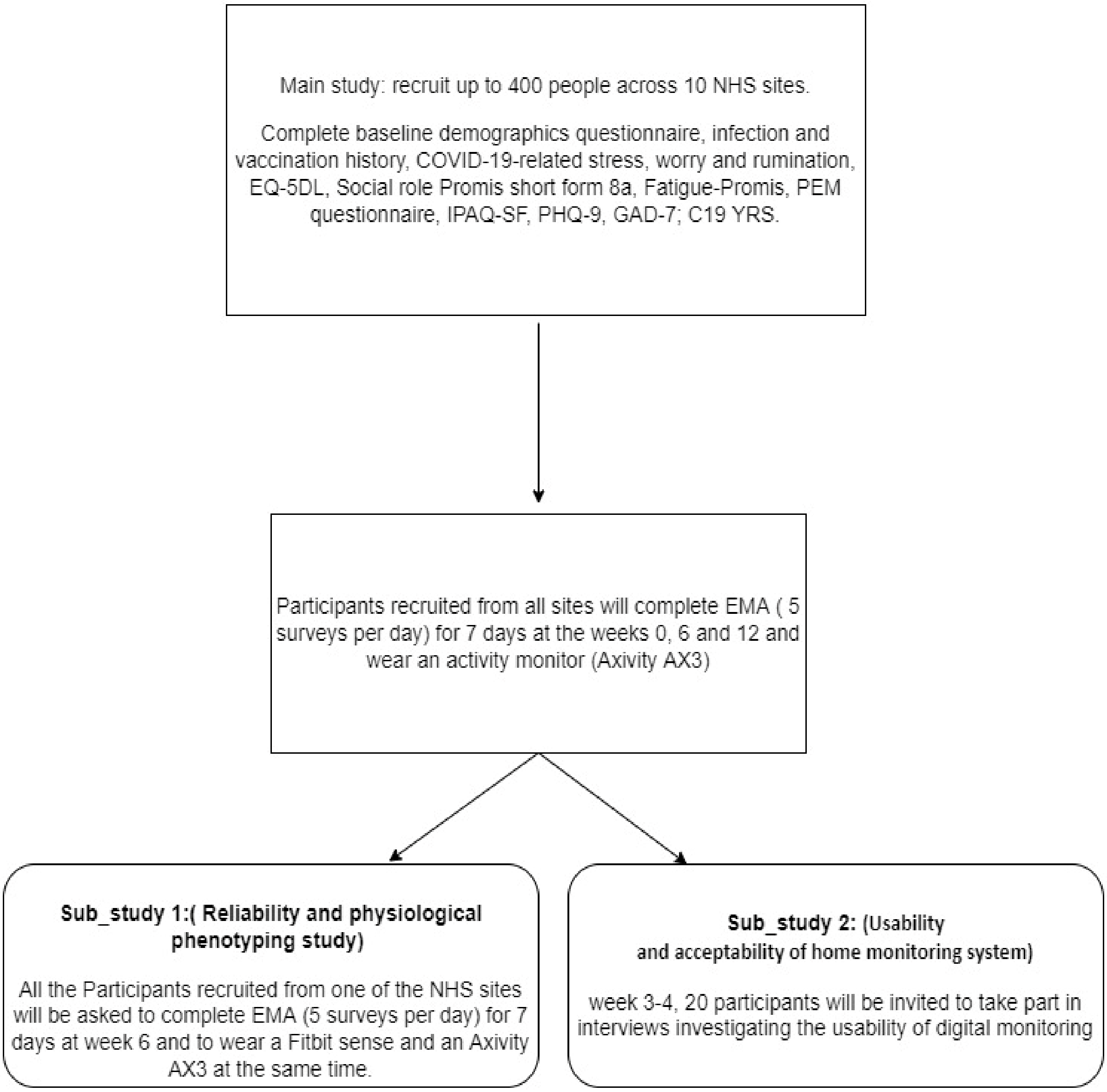

Figure 1. Main and sub-studies flowchart.

	Constructs	Scale	Questions	Timing
**1.Wellbeing**		NRS 11 point scale Sliding scale	**1. How well do you feel just now?**<br>Unwell – very well.	First survey of the day: suggest 9am but to be confirmed. Delivered 6 times randomly stratified between 10am and 8pm. Then at 9pm
**2.Sleep**	Quality<br>Amount /duration & how regular is sleep pattern?<br>Sleep latency (?)<br>Fractured sleep	4 point ordinal scale	**1. Thinking about your sleep last night, how would you rate the quality of your sleep?**<br>(Very good- very poor on sliding scale)<br>**2. What time did you go to bed last night?**<br>**3. What time did you get up this morning?**<br>**4. How long (in minutes) did it take you to fall asleep last night?**<br>(Options: 20 minutes or less, 20 to 60 minutes, 1-2 hours, more than 2 hours.)<br>**5. How many times did you wake up during the night?**<br>(Options: None, 1, 2-3, 4 or more)	First survey of the day: suggest 9am but to be confirmed
**3.Post exertion malaise**	PEM	1 hour or less, 1-3 hours, 4-8 hours, more than 8 hours.	**1. Thinking about today, did you experience worsening of**	Last survey of the day: suggest 9pm but to

For peer review only

			**your fatigue or Long Covid symptoms after minimal physical effort?** Yes/No<br><br>2. **Did you experience worsening of your fatigue or Long Covid symptoms after any mental effort?** Yes/No<br><br>3. **How long did this last?**<br><br>Options: 1 hour or less, 1-3 hours, 4-8 hours, more than 8 hours.	be confirmed.
**4.Activity**	Perceived effort Type – classified by mental/physical/social.	0 no effort -10 most effortful	1. **Thinking about what were you doing just before completing this survey, which one of the following best describes your activity:**<br><br>• Resting or sleeping<br>• Looking after myself (for example, getting dressed, eating)<br>• Mostly physical activity (for example walking, cleaning, exercising)	Delivered 6 times randomly stratified between 10am and 8pm. Then at 9 am and 9 pm

For peer review only

			• Mostly mental activity (such as reading, using a computer or phone, working or studying)<br>• Socialising or talking with other people<br><br>**2. How effortful was that activity?**<br><br>Sliding scale: 0 no effort to 10 most effortful	
**5.Psychological factors**	Stress<br>Repetitive thinking	Not at all/a Little/Moderately, Very.	**1. Thinking about the last hour, to what extent have you?**<br><br>• Felt stressed<br>• Worried about your illness in the future<br>• Thought about your illness in the past.	Delivered 6 times randomly stratified between 10am and 8pm.<br>Then at 9 am and pm
**6.Impact on daily functioning**	Severity and frequency?	Yes/no	**1. Thinking about the last hour have you experienced any of the following**<br><br>• Problems with personal care (washing/dressing)<br>• Problems doing usual activities<br>• Feeling exhausted after an activity	Delivered 6 times randomly stratified between 10am and 8pm.<br>Then at 9 am and pm

For peer review only

			• Problems walking about<br>• Problems communicating with others<br>• Problems concentrating or remembering things	
**7.Symptoms**	Frequency and severity		**1.** At the moment are you experiencing:<br><br>**Breathlessness** Yes/ no<br>If yes, it branches to the question: please rate how severe your breathlessness is:<br>***1 = Mild problem;***<br>***2 = Moderate problem;***<br>***3 = Severe problem;***<br><br>**2. Fatigue** yes/no<br><br>Branch as above.<br><br>**3. Pain/discomfort** yes/no<br><br>Branch as above.<br><br>**4. Dizziness or palpitations** yes/no<br><br>Branch as above.<br><br>**5. Feeling anxious** yes/no<br><br>Branch as above.<br><br>**6. Feeling depressed** yes/no<br><br>Branch as above.	Delivered 6 times randomly stratified between 10am and 8pm.<br>Then at 9 am and pm

For peer review only

**7.Change**	Improvement or decline in health status	Modified Patients' Global Impression of Change (PGIC)	See full assessment	onto redcap questionnai re
**8.Participatio n and activity**	Participation	Oxford Participation and Activities Questionnaire (OxPAQ)	See full assessment	onto redcap questionnai re
**9.Symptom burden and functional impact**	Multi dimensional	C19YRS	See full assessment	onto redcap questionnai re

Appendix 1: Ecological Momentary Assessment Ecological Momentary Assessment EMA assessment

For peer review only

BMJ Open

Digital home-monitoring for capturing daily fluctuation of symptoms; a longitudinal repeated measures study: Long Covid Multi-disciplinary Consortium to Optimise Treatments and Services across the NHS (A LOCOMOTION Study): a study protocol

Journal:	*BMJ Open*
Manuscript ID	bmjopen-2022-071428.R1
Article Type:	Protocol
Date Submitted by the Author:	27-Jun-2023
Complete List of Authors:	Mansoubi, Maedeh; University of Exeter<br>Dawes, Joanna; University of Exeter<br>Bhatia, Aishwarya; University of Exeter<br>Vashisht, Himanshu; Dublin City University<br>Collett, Johnny; Oxford Brookes University, Department of Sport, Health and Social Work<br>Greenwood, Darren; University of Leeds, Biostatistics Unit<br>Ezekiel , Leisle ; University of Southampton<br>O'Connor, Daryl; University of Leeds,<br>Leveridge, Phaedra ; University of Exeter<br>Rayner, Clare; University of Leeds<br>Read, Flo; University of Exeter<br>Sivan, Manoj; University of Leeds,<br>Tuckerbell, Ian; University of Leeds<br>Ward, Tomas; Dublin City University<br>Delaney, Brendan; Imperial College London, Surgery and Cancer<br>Muhlhausen , Willie; The Wild Research Limited<br>Locomotion consortium, Locomotion consortium; University of Leeds<br>Dawes, Helen; University of Exeter, medical school
**Primary Subject Heading**:	Rehabilitation medicine
Secondary Subject Heading:	Public health
Keywords:	Rehabilitation medicine < INTERNAL MEDICINE, Fatigue, PAIN MANAGEMENT, COVID-19

SCHOLARONE™
Manuscripts

For peer review only - http://bmjopen.bmj.com/site/about/guidelines.xhtml

Digital home-monitoring for capturing daily fluctuation of symptoms; a longitudinal repeated measures study: Long Covid Multi-disciplinary Consortium to Optimise Treatments and Services across the NHS (A LOCOMOTION Study): a study protocol

Authors:
Maedeh Mansoubi[1,2,3], Joanna Dawes[1], Aishwarya Bhatia[1], Himanshu Vashisht[4], Johnny Collett[5], Darren C. Greenwood[6], Leisle Ezekiel[7] , Daryl B. O'Connor[8], Phaedra Leveridge[1] Clare Rayner[9], Flo Read[1], Manoj Sivan[10], Ian Tucker-Bell[9], Tomas Ward[11], Brendan Delaney [12], Willie Muhlhausen[4], **LOCOMOTION consortium,** Helen Dawes[1,2,3]

Collaborators:
LOCOMOTION consortium: Nawar Diar Bakerly, Mauricio Barahona, Alexander Casson, Jonathan Clarke, Vasa Curcin, Helen Davies, Carlos Echevarria, Sarah Elkin, Rachael Evans, Zaccheus Falope, Ben Glampson, Trisha Greenhalgh, Stephen Halpin, Mike Horton, Joseph Kwon, Simon de Lusignan, Gayathri Delanerolle, Erik Mayer, Harsha Master, Ruairidh Milne, Ghazala Mir, Jacqui Morris, Amy Parkin, Stavros Petrou, Anton Pick, Nick Preston, Amy Rebane, Emma Tucker, Ana Belen Espinosa Gonzalez, Sareeta Baley, Annette Rolls, Emily Bullock, Megan Ball, Shehnaz Bashir, Joanne Elwin, Denys Prociuk, Iram Qureshi, Samantha Jones

Affiliation:

1. Medical School, University of Exeter, Exeter, UK
2. NIHR Exeter Biomedical Research Center, Medical School, Faculty of Health and Life sciences, University of Exeter, Exeter, EX1 2LU United Kingdom
3. Intersect@Exeter, Medical School, University of Exeter, Exeter, UK
4. The Wild Research Limited, Dublin, Ireland
5. Department of Sport, Health and Social Work, Oxford Brookes University
6. Academic Department of Rehabilitation Medicine, University of Leeds, Leeds, UK
7. Southampton University
8. School of Psychology, University of Leeds, UK
9. Patient Advisory Group (PAG) Representative
10. Faculty of Medicine and Health, School of Medicine, University of Leeds, UK
11. Insight SFI Research Centre for Data Analytics, Dublin City University, Dublin, Ireland
12. Faculty of Medicine, Department of Surgery & Cancer, Imperial College London

Corresponding Author
Helen Dawes
h.dawes@exeter.ac.uk
Medical school, University of Exeter
www.exeter.ac.uk
Medical School Building, College of Medicine and Health, St Lukes Campus, Heavitree Road, Exeter, EX12LU
Word count: 4289

For peer review only

Digital home-monitoring for capturing daily fluctuation of symptoms; a longitudinal repeated measures study: Long Covid Multi-disciplinary Consortium to Optimise Treatments and Services across the NHS (A LOCOMOTION Study)

Abstract

Introduction:

A substantial proportion of COVID-19 survivors continue to have symptoms more than three months after infection, especially of those who required medical intervention. Lasting symptoms are wide-ranging, and presentation varies between individuals and fluctuates within an individual. Improved understanding of undulation in symptoms and triggers may improve efficacy of healthcare providers and enable individuals to better self-manage their Long Covid. We present a protocol where we aim to develop and examine the feasibility and usability of digital home-monitoring for capturing daily fluctuation of symptoms in individuals with Long Covid and provide data to facilitate a personalised approach to the classification and management of Long Covid symptoms.

Methods and Analysis:

This study is a longitudinal prospective cohort study of adults with Long Covid accessing 10 National Health Service (NHS) rehabilitation services in the UK. We aim to recruit 400 people from participating NHS sites. At referral to study, six weeks and 12 weeks, participants will complete demographic data (referral to study) and clinical outcome measures, including Ecological Momentary Assessment (EMA) using personal mobile devices. EMA items are adapted from the COVID-19 Yorkshire Rehabilitation Scale (C19-YRS) items and include self-reported activities, symptoms, and psychological factors. Passive activity data will be collected through wrist-worn sensors. We will use latent class growth models to identify trajectories of experience, potential phenotypes defined by co-occurrence of symptoms, and interrelationships between stressors, symptoms, and participation in daily activities. We anticipate that n=300 participants provide 80% power to detect a 20% improvement in fatigue over 12 weeks in one class of patients relative to another.

Ethics and Dissemination:

The study was approved by the Yorkshire & The Humber—Bradford Leeds Research Ethics Committee (ref: 21/YH/0276). Findings will be disseminated in peer-reviewed publications and presented at conferences.

Trial registration number: ISRCTN15022307

Keywords: Long covid, Home monitoring, Ecological Momentary Assessment

For peer review only

Strengths and Limitations

- Recruitment of participants across multiple NHS Long Covid clinics across England.
- Readily available technology is used to monitor symptoms in real-time in the context of daily life.
- The reliability and validity of EMA constructs are limited.
- EMA completion rates and participant assessment reactivity is limited.
- The longitudinal and remote monitoring aspect of the study means there is a risk of some participants not engaging with the study and participant attrition.

For peer review only

INTRODUCTION

Since the start of the COVID-19 pandemic in 2019, over 480 million people across the globe have had confirmed COVID-19 infection (1). A substantial proportion of COVID-19 survivors will continue to have symptoms more than three months after infection, especially, but not restricted to, those requiring medical intervention for the infection (2–5). The term Long COVID is used to define the long-lasting symptoms of COVID-19 and includes ongoing symptomatic COVID-19 (from 4 to 12 weeks) and post-COVID-19 syndrome (12 weeks or more) (6). In the UK alone, an estimated 1.5 million (5) and over 100 million people globally (2) are living with self-reported Long Covid (5).

The presentation of Long Covid is variable with a wide range of symptoms which most commonly include general weakness, malaise, fatigue, breathlessness, and impaired concentration (7–9). Long Covid has been described as having "undulating symptoms" where individuals may experience feeling well but then have a re-occurrence of symptoms, sometimes in response to triggers such as stress or physical activity (10). The need for a greater degree of definitional stringency and clearer classification of symptom incidences, number, severity, trajectory and duration for clinical trials has been highlighted (11). To address this, and a need to improve access to multi-disciplinary health care for the large numbers of people reporting Long Covid (10), we propose exploring the use of remote monitoring methods with readily available technology as a potential tool to assist patients and healthcare professionals in the clearer classification of symptoms and a better understanding of symptom management and help with recovery (12,13).[1]

There are currently no validated monitoring systems to classify and regularly check a patient's symptoms and report them to doctors and other healthcare professionals. A recent systematic review including 272 articles demonstrated that remote patient monitoring methods could improve patient support and the efficiency of the therapy (14). According to this review, smartphone applications and wireless devices are the most common Remote Patient Monitoring (RPM) methods (14). Given the lack of evidence on a valid and feasible method to support patients and health systems with COVID symptom RPM and management, this work package of the LOCOMOTION study (15) aims to develop and examine the feasibility and usability of home monitoring (using

[1]Abbreviations:

Long Covid Multi-disciplinary consortium to Optimise Treatments and services across the NHS (LOCOMOTION), COVID-19 Yorkshire Rehabilitation Scale (C19-YRS), Remote Patient Monitoring (RPM), Post-exertional malaise (PEM), University of California, San Diego Brief Assessment of Capacity to Consent Brief Assessment of Capacity to Consent (UBACC), Research Electronic Data Capture (REDCap), Vector Autoregressive modelling (VAR), Markov chain Monte Carlo (MCMC), Ecological Momentary Assessment (EMA).

For peer review only - http://bmjopen.bmj.com/site/about/guidelines.xhtml

smartphone applications and wearable sensors) for capturing the daily fluctuation of symptoms in real-time, recognising adverse triggers across patients from a range of demographic groups and aid self-management.

The primary objectives of the study are:

1. To quantify the extent to which total (per day and week) and specific (at each time point) cognitive, physical, and social activities, sleep and rest predict subsequent incidence, severity, number and duration of Long Covid symptoms, including post-exertional malaise (PEM). By total activities, this project refers to the overall engagement in cognitive, physical, and social activities over a given period. Specific activities encompass the individual types of cognitive, physical, and social activities conducted at each specific time point.
2. To identify different groups or classes of people who respond to physical or mental effort (based on their response to questionnaires, app surveys and accelerometer result) differently in regards to PEM, other symptom incidences, duration, number and severity. Responding to physical or mental effort relates to the participant's response or experience following engagement in activities that require physical or mental exertion. This can include fatigue, symptom exacerbation, or any other subjective or objective measures of response to effort.

The secondary objectives of the study are to:

3. Quantify the extent to which the prevalence or severity of specific symptoms predicts subsequent activity.
4. Quantify the relationship between sleep duration and quality with Long Covid symptoms.
5. Explore the relationship between different component contributions to the overall level of physical activity (frequency, intensity, recovery, duration) and subsequent symptoms.
6. Explore the associations between different timing or degree of exposure to (cognitive, physical and social) activities in terms of cumulative exposure (e.g. multiple triggers in a short space of time or repeated triggers over the day), along with the time-lagged onset of symptoms or exacerbation of symptom severity.
7. Determine the extent of the relationship between physiological variables and activity and symptom severity for different groups of activities and symptoms. (Substudy 1).
8. Describe the stability of the activity, symptoms and the interrelationship in people with Long Covid.
9. Describe the variability in activities, dose of activities and symptom severity

For peer review only

10. Explore the moderating effects of living with physical health and/or mental health conditions (including depression and anxiety) on the relationships between the triggers and symptoms of Long Covid (as outlined in objectives 1 and 2).
11. Describe the concurrent validity of components of the home monitoring system (Substudy 1)
12. Investigate usability and acceptability of home monitoring system and explore patient experiences of Long Covid (Substudy 2)

METHODS AND ANALYSIS

This is Workstream 2.1 of the LOCOMOTION Health Service Study (Trial Registration Number ISRCTN15022307) (15). The objective of this workstream is to specifically focus on the remote monitoring of individuals with long covid. It aims to assess the effectiveness of remote monitoring techniques in tracking and managing the symptoms of long covid. The study follows Strobe guidelines https://www.equator-network.org/reporting-guidelines/strobe/.

Design and setting: The study planned start date is February 2022, and the anticipated end date, is September 2023. Using a longitudinal study design with repeated measures and a nested sub-study, we aim to recruit a purposive sample of patients with (self-reported/GP diagnosed) Long Covid who have been referred to one of the ten participating NHS rehabilitation clinics (see figure 1). We will recruit 400 participants with the anticipation of a 25% dropout, giving 300 patients (40 per site) using purposive sampling.

At referral to study, at six weeks, and 12 weeks, participants will complete demographic data (on referral to study) and clinical outcome measures, including Ecological Momentary Assessment (EMA) and passive activity data over seven days using personal mobile devices. EMA symptoms items are adapted from the COVID-19 Yorkshire Rehabilitation Scale (C19-YRS) items and include self-reported activities, symptoms and psychological factors. Passive activity data will be collected through wrist-worn sensors. EMA consists of sampling an individual's experiences and subjective states as they occur in real-time in the individual's natural environment (16). There will be two embedded sub-studies. Sub-study 1 will include additional home digital physiological measures of heart rate variability (HRV) to test biofeedback (improving symptoms by increasing HRV). Sub-study 2 will use qualitative interviews to assess the usability and acceptability of using home monitoring (via smartphones) of triggers and symptoms of Long Covid at clinic appointments and explore patient experiences of Long Covid.

Inclusion criteria

We will include all adults who are 18 years and over, able to participate in the study (i.e., able to complete the EMA and use the wearable technology), able to give informed consent and have

For peer review only

English language proficiency to the level of conducting health consultations. Participants will not be included or excluded based on past or current illness or medication at the time of the presumed Covid infection; however, they will be asked to describe these at the start of this study.

Exclusion criteria

Participants will be excluded who are under 18 years old, unable to use the EMA app technology or use the wearable technology, unable to understand the language used in the EMA or are known to be pregnant. We will also exclude those with a known previous diagnosis of dementia or cognitive impairment that would prevent ability to participate in the use of the EMA or wearable technologies.

Main study

The main LOCOMOTION study, encompassing all workstreams, aims to recruit a total of 5,000 participants. Within this comprehensive study, workstream 2.1 specifically focuses on recruiting 400 individuals with Long Covid. This dedicated workstream aims to explore and evaluate remote monitoring techniques for managing and tracking the symptoms of Long Covid. By recruiting this specific cohort, we aim to gather valuable insights and data to enhance the understanding and treatment of Long Covid within the broader LOCOMOTION study. With broad assumptions, based on pilot data from our rehabilitation clinics, we anticipate n=300 participants would provide approximately 80% power to detect 20% improvement in fatigue over 12 weeks in one class of patients relative to another, e.g., a stable symptom group with fatigue scores of 5 at each time point, compared to a group gradually improving from 5 to 4, with within-person correlation 0.7, residual variance 2.0, and 30% with the symptom pattern. We anticipate up to 25% dropout across follow-up, so we initially aim to recruit up to n=400.

Sub study 1: (Reliability and physiological phenotyping study of home monitoring system)

This study will invite 50 participants, allowing for dropouts and leaving a sample size of approximately 30 participants to ensure meeting central limit theorem criteria for reliability analysis and between 25-50 for meeting feasibility criteria (see (17))

Sub study 2: (Usability and acceptability of home monitoring system)

This study will invite 20 patients who have taken part in the main monitoring study to be interviewed and 10 clinicians working in one of the collaborating Long Covid clinics. This sample size should ensure sufficient 'information power' to address the research questions (18).

Recruitment

Recruit Each of the ten participating Long Covid clinics has a clinical research lead who is responsible for inviting patients to participate in the study on referral. Potential participants will be notified of

For peer review only

the opportunity to take part in the study by the clinical research lead at each site and provided with a link to an online form to register their expression of interest and check that they meet the study inclusion criteria. Intake will be capped when recruitment has achieved the study sample size, and further applicants will receive an email response that the study is closed but that they will be contacted again when it is open.

Data collection

Baseline and follow-up data collection

Baseline and follow-up measures will be collected using the online data capture platform REDCap. On entry to the study, participants will also complete the C19-YRS and provide information about their demographics (age, height, weight, sex, ethnicity, postcode, date of birth, marital status, housing, education, employment, smoking, disability, past medical history, drug history, covid history and care). Additionally, at weeks one, six and twelve, participants will complete the COVID-19 Yorkshire Rehabilitation Scale (C19-YRS) (19) and provide information about their physical activity levels, fatigue, quality of life and participation, wellbeing, anxiety and rumination, worry, depression and post-exertional malaise.

Wearables for home monitoring of fluctuation of symptoms

At referral to the study, at six weeks and 12 weeks, participants will be asked to perform ecological momentary assessments (EMA) over 7 days using personal mobile devices and wearable sensors and accelerometers. The EMA is delivered using the AthenaCX platform, a secure and GDPR-compliant research survey platform (20) which works on Apple and android phones. The EMA includes five time-contingent surveys (sent between 8.45 am and 9.15 pm) of self-reported activities, symptoms and psychological factors (see Appendix 1). EMA questions were adapted from the C19-YRS items to enable home monitoring of symptom fluctuation and trigger identification, and from items developed for the UK COVID-19 Mental Health and Wellbeing study (21). Pilot work has demonstrated that this approach is feasible and acceptable to Long Covid patients.

Accelerometer sensor

The daily amount of physical activity and sedentary time will be objectively assessed using the Axivity AX3, a “wrist-worn” tri-axial accelerometer designed by Open Lab, Newcastle University, UK. AX3 data will be downloaded using OMGUI software (open movement [V.1.0.0.37]). The Axivity AX3 is a valid and reliable method for the measurement of physical activity and sedentary behaviour (22)

Sub study 1 (Reliability and physiological phenotyping study of home monitoring system):

For peer review only

Reliability and physiological phenotyping study of 50 patients in Oxford to include additional home digital physiological measures HRV (heart rate variability)

Method: Fifty patients from the clinic in Oxford will be invited to participate in the following voluntarily and will be able to opt-in or out of any of the components at each testing time point: digitally and remotely monitoring physiological measures via a wrist-worn or a chest strap heart rate sensor, based on participants' preference and capability to measure heart rate and heart rate variability. This aims to explore the potential to use measuring heart rate and heart rate variability as feedback for clinical purposes.

Sub-study 2 process evaluation (Usability and acceptability of home monitoring system):

Usability, acceptability and co-design qualitative study with 20 patients and ten clinicians and a clinician survey to assess the usability and acceptability of using home monitoring (via smartphones) of triggers and symptoms of Long Covid at clinic appointments and explore patient experiences of Long Covid.

Method: Sub-study 2 will be a co-design study between 20 patients and ten clinicians. It will use a survey to explore symptom behaviour and establish the utility of prospective symptom fluctuation and triggers for self-management. We will conduct a short debrief interview with up to 20 participants during either week three or week four of their study. This will be conducted by phone/video call and recorded. The interview will be structured around the usability dimensions of efficiency, effectiveness, and satisfaction. In particular, the interview will ask about both the apps and using them in daily life, any issues identified and any suggestions for improvement to the app or the study design. The interview will include a "would you recommend to someone you know?" question and explore reasons for this. The interview will also include a think-aloud component in which the interviewer asks the participant to go through a data entry cycle and describe what they are thinking or doing as they go along. Towards the end of the interview, the interviewee will be asked to complete the 10-item System Usability Scale. Participants will also be interviewed about the experiences of Long Covid.

For clinicians, the acceptability and usability assessment will focus on content, design, language and adaptability of daily monitoring information in the clinic environment and explore views on the usefulness of this approach to support Long Covid patients. Semi-structured interviews will be conducted either face to face or via the secure web video conferencing system.

For peer review only

Data cleaning

Reported values more than 3 standard deviations from the mean will be double-checked with original data sources. Observations with Studentised residuals greater than 3, Cook's distance greater than 1, and leverage of $h>0.3$ will be used to detect influential data points in subsequent models. No data will be excluded without evidence of error or implausibility, other than through sensitivity analyses.

Descriptive statistics

Patient characteristics (demographics, clinical history, exposures and outcomes) will be tabulated overall, by sex, by 2 to 5 broad age bands derived from the data to provide similar numbers in each age group, and by 2 to 5 fatigue bands, similarly derived, with sensible rounding of thresholds (e.g. to nearest 5 years or to nearest 1 point on scale).

Visual analogue scales and measures will be presented as median and interquartile range or mean and standard deviation, dependent on the underlying distributions of the scores.

The characteristics of consenting participants will be compared with the characteristics of all clinic patients, to investigate whether the participating patients differ systematically from those not participating. Characteristics of participants with incomplete data will be compared to those with complete data.

Statistical modelling

Primary objective 1:

Multilevel multivariate vector autoregressive (VAR) modelling will be used to investigate the associations between C19-YRS symptom scores, and the activities reported in the EMA, taking account of the hierarchical/nested multilevel structure of the data (Objective 1). We will construct multilevel multivariate vector autoregressive models within the Dynamic Structural Equation Model (DSEM) module in Mplus 8.6 or WinBUGS 1.4 to partition total variability across individuals and time points into within- and between-person components. We anticipate using non-informative priors and two independent Markov chain Monte Carlo (MCMC) chains, allowed to burn in until distributions are stable.

The within-person component will be modelled using a vector autoregressive (VAR) time-series model of order 1. This model will be used to predict the outcomes at one-time point

For peer review only

using all predictor variables at the previous time point. This will allow estimation of the within-person associations between symptom outcomes and potential triggers, the autoregressive effects that the severity of the outcome variable has on itself at the next time point, and the cross-lagged effects of one variable on another variable at the next time point. Both autoregressive and cross-lagged parameters will be modelled as random effects that vary across individuals. We anticipate fitting both random intercepts and slopes but assuming these are independent of each other. Robustness to this assumption will be assessed in sensitivity analyses, where sufficient degrees of freedom and complexity of models allow.

We will estimate the association of between-individual differences in the random-effects with variation in other variables measured at the between-person level, e.g. whether change symptoms over time are associated with within-person dynamics between symptom severity and potential triggers such as activities and effort, by specifying the change in symptom severity as the outcome variable and the random effects of the cross-lagged and autoregressive parameters as predictors. All variables and parameters will be centred, standardised or detrended where appropriate to facilitate correct analysis and interpretation.

To allow for both shorter and longer-term lag effects between triggers and outcomes, analyses will be conducted first with each EMA as the time point (e.g. to compare mental effort at one assessment with fatigue at the next), then repeated using the whole day as the timepoint, aggregating over the 6 EMAs that day (e.g. to compare impact of physical effort one day on symptoms the next day). This will also be used for any outcomes only measured once in a day.

The same methods will be applied to secondary objective 3 (symptoms predicting subsequent activity), objective 4 (timing, duration and quality of sleep), objective 5 (components of physical activity), objective 6 (timing and cumulation of activities), and in part to objectives 7 (heart rate variability and physiological phenotyping) and 8 (stability of association).

Primary objective 2:

Latent class growth models will be used to identify different types of symptom trajectories experienced by those living with Long Covid, potential phenotypes defined by co-occurrence of symptoms, and any interrelationships between triggers, symptoms, and participation in daily activities, personal care such as washing or dressing, usual activities, problems walking about, problems communicating with others, problems with concentration or remembering things (Objective 2). The extent of these between-person and within-person day-to-day fluctuations will be quantified, and the potential for characteristics to predict subsequent

For peer review only

improvement over time. The number of classes will be based on information criteria (e.g. BIC and AIC) and clarity of interpretation of the domains identified.

Functional data analysis will also be used, where appropriate, to characterise features of long-term symptom trajectories, such as variation and speed of change. The extent of these between-person and within-person day-to-day fluctuations will be quantified, and the potential for characteristics to predict subsequent improvement over time.

These methods will also be applied in part to objectives 7 (heart rate variability and physiological phenotyping) and 8 (stability of association).

All statistical analyses will be conducted using SPSS 28, R, Stata 17 and MPlus 8.6 or WinBUGS 1.4.

Bias

Potential confounding

Potential confounders will be identified based on directed acyclic graphs, but as the aim is prediction, we will also include potential competing exposures in models.

Missing and incomplete data

The latent class growth models utilise mixed-effects modelling that allows for partially incomplete repeated measures across participants.

Exclusions, sensitivity analyses and subgroup analyses

Sensitivity analyses will be conducted to assess robustness to different types of trajectories for men vs women, hospitalised vs non-hospitalised, individuals with existing mental health conditions vs individuals without existing mental health conditions, different waves of acute infection as a proxy for variant, broad age band.

We have previously described sensitivity analysis assessing robustness of the models to the timeframe used for the VAR analysis, i.e. EMA or summary measure over the whole day We will also assess robustness of the model to 2nd order time-lags, in addition to the 1st order time-lags, where the data allows.

We assume independence of random intercepts and slopes but will assess robustness to this correlation structure where the data allows.

As sensitivity analysis to the effects of incomplete data, we will repeat excluding any days with fewer than 2 EMAs.

For peer review only

Thematic analysis

Thematic content analysis of interview transcripts will be used to investigate the usability and perceived usefulness of the digital measurement tools (by clinicians and patients) to support self-management and rehabilitation. It will be also used to explore participants' experiences of Long Covid triggers and symptoms. All qualitative data collection for the process evaluation will be carried out by a research team member with qualitative research experience. Interviews will be transcribed verbatim and analysed using thematic analysis methods which involves familiarisation with the interview, coding, developing and applying an analytical frame-work, charting data into the analytical framework for analysis (23). Interview transcripts will be coded using NVivo software (or similar software). Each interview will be independently coded by two reviewers. After coding four transcripts, reviewers will compare codes and discrepancies will be discussed and resolved prior to coding the remaining transcripts. The interim analysis will be conducted following an initial sample of 150 patients to determine whether saturation of themes had been reached. Mean (±standard deviation) or median and interquartile range values will be used as appropriate to summarise participants' demographic data.

Interviews will be analysed thematically initially to explore usability factors. Due to the focus of this process evaluation, the personal documentation will be initially subjected to content analysis—with a more detailed thematic analysis applied if required. Data will be reviewed in light of any contradictions and will guide a member-checking process with participants, which will be conducted prior to the write-up and dissemination of findings. Findings will be separated into the process and measure ready for reporting, but with consideration being given to where/if the doing of any process is a major contributor to the outcomes or perceived experience of participants. At this point, we will explore possible mechanisms for any observed effects, to generate change objectives, and behavioural outcomes.

Public patient involvement and engagement

The team have regular public patient involvement (PPI) meetings, and the study was designed in consultation with Long Covid patients on how COVID-19 has disproportionately affected different communities. The LOCOMOTION study has a seven-member core PPI Advisory Group (PAG) that includes different cultural, ethnic, and socioeconomic groups. They have contributed to the proposal research planning meetings and met separately to examine and develop the research aim,

For peer review only

objectives, and questions ensuring these align with the key research priorities of patients with Long Covid and represent different patient needs.

Ethics and Dissemination

The study was approved by the Yorkshire & The Humber—Bradford Leeds Research Ethics Committee (ref: 21/YH/0276) and conforms with the Declaration of Helsinki.

Participants consent to participate following an e-consent process. The e-consent form will be implemented through a secure web-based online survey accessible by computer, mobile phone, or tablet. The e-consent process also includes questions about the person's understanding of the study based on the Brief Assessment of Capacity to Consent (UBACC) (24); participants need to answer at least 6 of 8 questions correctly to be eligible for the study. This process queries the person's understanding of the consent form and has been used in over 200 research studies worldwide. A parallel consent process using telephones is available for those participants who are not able to use, or do not have access to the internet. Potential participants will be telephoned when the clinical research lead indicates this is the preferred mode of contact and communication to the research team. A call will be set up for one of the research teams to provide them with information. If the potential participant is interested, a second call will elicit their consent over the phone using the same script as e-consent and conduct the screening process using the same questionnaire. The questions on the capacity to consent quiz will be asked orally.

Findings will be disseminated through manuscript publications in peer-reviewed journals and conference presentations.

DISCUSSION

The overarching aim of this study is to optimise the classification and self-management of Long Covid by tracking symptoms in real-time in the context of daily life, and by using a data-driven approach to identify adverse triggers of symptoms and phenotypes of Long Covid. These findings also aim to extend knowledge of the trajectory of Long Covid, particularly interactions between Long Covid symptoms and activity-related behaviours. Understanding the relationship between Long Covid symptoms and everyday activity is essential for supporting effective self-management of Long Covid, particularly on achieving balance of activity, priority of activity in order to manage social, family and work commitments. The study findings will help facilitate a personalised approach to the management of Long Covid based on ecologically valid self-report data.

Additionally, we will evaluate the usability of digital home monitoring by patients and clinicians. Taken together the findings of these studies will help support the potential development of a digital

For peer review only

home monitoring, self-management platform for use with Long Covid patients. It is hoped that a future home monitoring platform will enable the provision of cost and time-effective care.

Strengths and limitations

Strengths

Key strengths of this study are the planned recruitment of participants through multiple NHS Long Covid clinics across England, and the use of readily available technology and real-time monitoring of symptoms in the context of daily life. These aspects of the study design increase the generalizability and ecological validity of the study findings.

Limitations

There are several known methodological limitations to ecological momentary assessment: the reliability and validity of EMA constructs, EMA completion rates and participant assessment reactivity (25). In addition, the longitudinal and remote monitoring aspect of the study means there is a risk of some participants not engaging with the study, and of participant attrition. We will use several strategies to minimise these limitations. Firstly, we optimised the face and construct validity of the EMA questions by using single constructs items from the C19-YRS (19) and PEM (26). In addition, the EMA was tested by people with Long Covid and clinicians with expertise in Long Covid to check the clarity and relevance of questions. We also tested the EMA schedule with people with Long Covid to ensure the schedule was acceptable and to optimise completion rates. We planned bursts of assessment (7 days repeated three times) rather than extended periods, thereby reducing the burden on the participant. Finally, we recognise the need to support participants through the first stages of using the EMA app; therefore, each participant will have an initial phone call from a member of the research team and subsequently have ongoing contact with the team via email or telephone.

Author contributions

All the below contributed to study design, drafting editing or revising paper, and have given final approval and agreed to accountability.

Maedeh Mansoubi, Joanna Dawes, Johnny Collett, Leisle Ezekiel, and Helen Dawes, Brendan Delaney, Darren C. Greenwood, Daryl B. O'Connor, Clare Rayner, Manoj Sivan, Ian Tucker-Bell, Tomas Ward, Himanshu Vashisht, Aishwarya Bhatia Flo Read, Phaedra Leveridge and Willie Muhlhausen.

LOCOMOTION consortium authors contributed in the protocol design.

For peer review only

Funding statement: This project has been funded by NIHR, grant reference number which is: COV-LT2-0016, Trial registration number NCT05057260, ISRCTN15022307. This work was supported by NIHR grant reference number [COV-LT2-0016].

Acknowledgement: This study was funded/supported* by the National Institute for Health and Care Research Exeter Biomedical Research Centre. Professor Helen Dawes and Dr Maedeh Mansoubi's work is supported by the National Institute for Health and Care Research Exeter Biomedical Research Centre. Dr Johnny Collett is funded by the NIHR Oxford Health Biomedical Research Centre. The views expressed are those of the author(s) and not necessarily those of the NIHR or the Department of Health and Social Care.

Competing interests statement: All authors declare that they have no conflicts of interest.

For peer review only

REFERENCES

1. WHO. WHO Coronavirus (COVID-19) Dashboard [Internet]. 2022 [cited 2022 Sep 23]. Available from: https://covid19.who.int/

2. Chen C, Haupert SR, Zimmermann L, Shi X, Fritsche LG, Mukherjee B. Global Prevalence of Post-Acute Sequelae of COVID-19 (PASC) or Long COVID: A Meta-Analysis and Systematic Review. J Infect Dis. 2022;19. DOI: 10.1093/infdis/jiac136

3. Fernández-de-las-Peñas C, Palacios-Ceña D, Gómez-Mayordomo V, Florencio LL, Cuadrado ML, Plaza-Manzano G, et al. Prevalence of post-COVID-19 symptoms in hospitalized and non-hospitalized COVID-19 survivors: A systematic review and meta-analysis. Eur J Intern Med. 2021;92(May):55–70. DOI: 10.1016/j.ejim.2021.06.009

4. Sugiyama A, Miwata K, Kitahara Y, Okimoto M, Abe K, E B, et al. Long COVID occurrence in COVID-19 survivors. Sci Rep. 2022;12(1):1–11. DOI: 10.1038/s41598-022-10051-z

5. ONS. Prevalence of ongoing symptoms following coronavirus (COVID-19) infection in the UK: 1 September 2022 [Internet]. 2022 [cited 2022 Sep 1]. Available from: https://www.ons.gov.uk/peoplepopulationandcommunity/healthandsocialcare/conditionsanddiseases/bulletins/prevalenceofongoingsymptomsfollowingcoronaviruscovid19infectionintheuk/1september2022

6. NICE. COVID-19 rapid guideline: managing the long-term effects of COVID-19 [Internet]. 2021 [cited 2022 Mar 29]. Available from: https://www.nice.org.uk/guidance/ng188

7. Aiyegbusi OL, Hughes SE, Turner G, Rivera SC, McMullan C, Chandan JS, et al. Symptoms, complications and management of long COVID: a review. J R Soc Med. 2021;114(9):428–42. DOI: 10.1177/01410768211032850

8. Raveendran A V., Jayadevan R, Sashidharan S. Long COVID: An overview. Diabetes Metab Syndr Clin Res Rev. 2021;15(3):869–75. DOI: 10.1016/j.dsx.2021.04.007

9. Michelen M, Manoharan L, Elkheir N, Cheng V, Dagens A, Hastie C, et al. Characterising long COVID: A living systematic review. BMJ Glob Health. 2021;6(9):1–12. DOI: 10.1136/bmjgh-2021-005427

10. NIHR. Living with Covid19 – Second review [Internet]. 2021 [cited 2021 Mar 1]. Available from: https://evidence.nihr.ac.uk/themedreview/living-with-covid19-second-review/

11. Ceban F, Leber A, Jawad MY, Yu M, Lui LMW, Subramaniapillai M, et al. Registered clinical trials investigating treatment of long COVID: a scoping review and recommendations for research. Infect Dis. 2022;54(7):467–77. DOI: 10.1080/23744235.2022.2043560

12. O'Connor DB, Aggleton JP, Chakrabarti B, Cooper CL, Creswell C, Dunsmuir S, et al. Research priorities for the COVID-19 pandemic and beyond: A call to action for psychological science. Br J Psychol. 2020;111(4):603–29. DOI: 10.1111/bjop.12468

13. Chiauzzi E, Rodarte C, DasMahapatra P. Patient-centered activity monitoring in the self-management of chronic health conditions. BMC Med. 2015;13(1):1–6. DOI: 10.1186/s12916-015-0319-2

For peer review only

14. de Farias FAC, Dagostini CM, Bicca Y de A, Falavigna VF, Falavigna A. Remote Patient Monitoring: A Systematic Review. Telemed E-Health. 2020;26(5). DOI: 10.1089/tmj.2019.0066

15. Sivan M, Greenhalgh T, Darbyshire JL, Mir G, O'Connor RJ, Dawes H, et al. LOng COvid Multidisciplinary consortium Optimising Treatments and servIces acrOss the NHS (LOCOMOTION): protocol for a mixed-methods study in the UK. BMJ Open. 2022;12(5):e063505. DOI: 10.1136/bmjopen-2022-063505

16. Shiffman S, Stone AA, Hufford MR. Ecological momentary assessment. Annu Rev Clin Psychol. 2008;4:1–32. DOI: 10.1146/annurev.clinpsy.3.022806.091415

17. NIHR. Justify sample size for a feasibility study [Internet]. [cited 2022 Mar 1]. Available from: https://www.rds-london.nihr.ac.uk/resources/justify-sample-size-for-a-feasibility-study/

18. Malterud K, Siersma VD, Guassora AD. Sample Size in Qualitative Interview Studies: Guided by Information Power. Qual Health Res. 2016;26(13):1753–60. DOI: 10.1177/1049732315617444

19. O'Connor RJ, Preston N, Parkin A, Makower S, Ross D, Gee J, et al. The COVID-19 Yorkshire Rehabilitation Scale (C19-YRS): Application and psychometric analysis in a post-COVID-19 syndrome cohort. J Med Virol. 2022;94(3):1027–34. DOI: 10.1002/jmv.27415

20. AthenaCX. AthenaCX [Internet]. [cited 2022 Nov 6]. Available from: https://www.athenacx.com/

21. O'Connor DB, Wilding S, Ferguson E, Cleare S, Wetherall K, McClelland H, et al. Effects of COVID-19-related worry and rumination on mental health and loneliness during the pandemic: longitudinal analyses of adults in the UK COVID-19 mental health & wellbeing study. J Ment Health. 2022;0(0):1–12. DOI: 10.1080/09638237.2022.2069716

22. De Craemer M, Verbestel V. Comparison of outcomes derived from the actigraph GT3X+ and the axivity AX3 accelerometer to objectively measure 24-hour movement behaviors in adults: A cross-sectional study. Int J Environ Res Public Health. 2022;19(1):4–11. DOI: 10.3390/ijerph19010271

23. Braun V, Clarke V. Using thematic analysis in psychology. Qual Res Psychol. 2006;3(2):77–101.

24. NICE. Decision-making and mental capacity [Internet]. 2018 [cited 2022 Apr 23]. Available from: https://www.nice.org.uk/guidance/ng108

25. Ram N, Brinberg M, Pincus AL, Conroy DE. The Questionable Ecological Validity of Ecological Momentary Assessment: Considerations for Design and Analysis. Res Hum Dev. 2017;14(3):253–70. doi: 10.1080/15427609.2017.1340052

26. Cotler J, Holtzman C, Dudun C, Jason L. A Brief Questionnaire to Assess Post-Exertional Malaise. Diagnostics. 2018;8(3):66. doi: 10.3390/diagnostics8030066

For peer review only

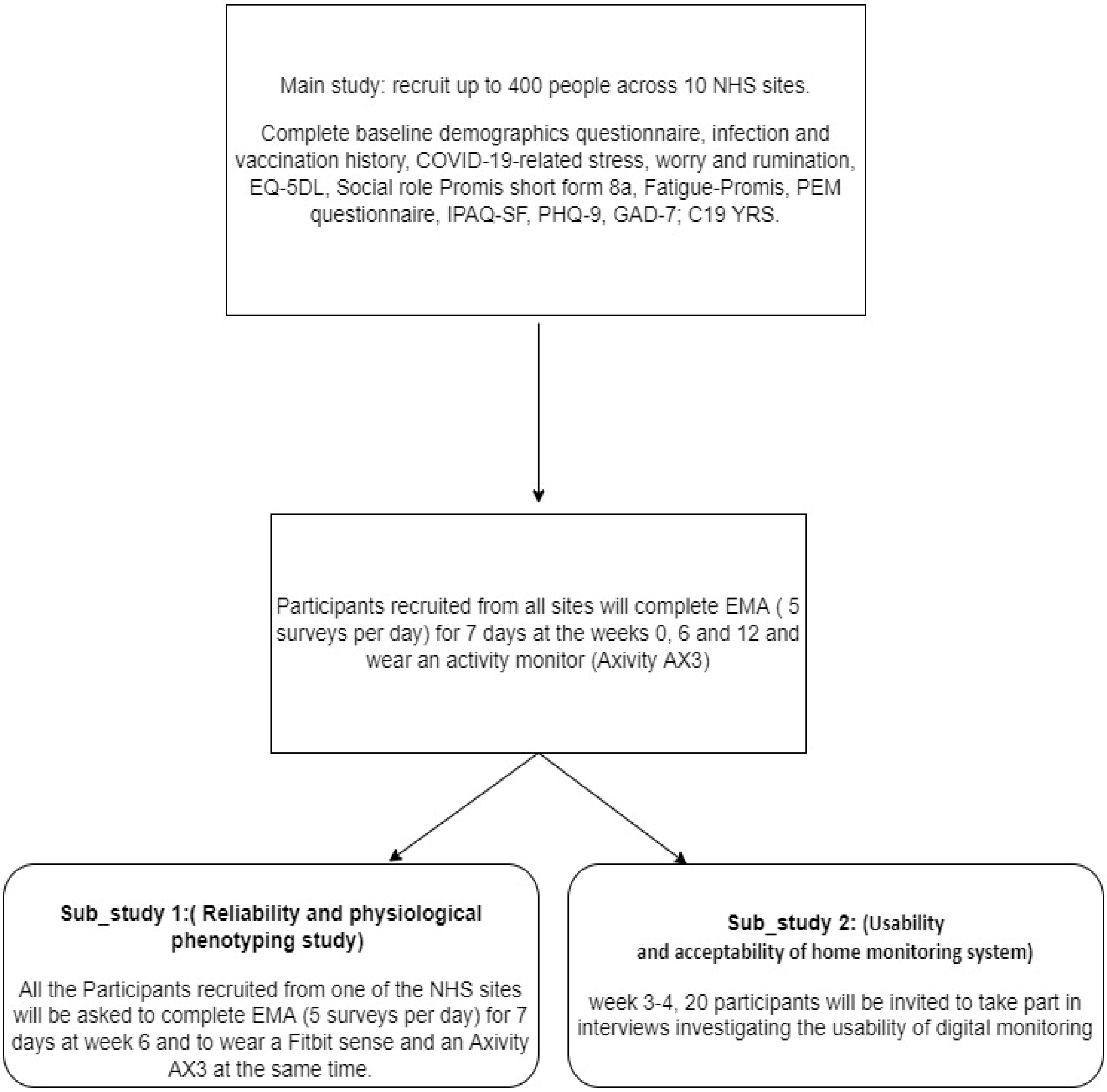

Figure 1. Main and sub-studies flowchart.

	Constructs	Scale	Questions	Timing
**1.Wellbeing**		NRS 11 point scale Sliding scale	**1. How well do you feel just now?**<br><br>Unwell – very well.	First survey of the day: suggest 9am but to be confirmed. Delivered 6 times randomly stratified between 10am and 8pm. Then at 9pm
**2.Sleep**	Quality<br>Amount /duration & how regular is sleep pattern?<br>Sleep latency (?)<br>Fractured sleep	4 point ordinal scale	**1. Thinking about your sleep last night, how would you rate the quality of your sleep?**<br><br>(Very good- very poor on sliding scale)<br>**2. What time did you go to bed last night?**<br>**3. What time did you get up this morning?**<br>**4. How long (in minutes) did it take you to fall asleep last night?**<br><br>(Options: 20 minutes or less, 20 to 60 minutes, 1-2 hours, more than 2 hours.)<br>**5. How many times did you wake up during the night?**<br><br>(Options: None, 1, 2-3, 4 or more)	First survey of the day: suggest 9am but to be confirmed
**3.Post exertion malaise**	PEM	1 hour or less, 1-3 hours, 4-8 hours, more than 8 hours.	**1. Thinking about today, did you experience worsening of**	Last survey of the day: suggest 9pm but to

For peer review only

			**your fatigue or Long Covid symptoms after minimal physical effort?** Yes/No<br><br>2. **Did you experience worsening of your fatigue or Long Covid symptoms after any mental effort?** Yes/No<br><br>3. **How long did this last?**<br><br>Options: 1 hour or less, 1-3 hours, 4-8 hours, more than 8 hours.	be confirmed.
**4.Activity**	Perceived effort Type – classified by mental/physical/soci al.	0 no effort -10 most effortful	1. **Thinking about what were you doing just before completing this survey, which one of the following best describes your activity:**<br><br>• Resting or sleeping<br>• Looking after myself (for example, getting dressed, eating)<br>• Mostly physical activity (for example walking, cleaning, exercising)	Delivered 6 times randomly stratified between 10am and 8pm. Then at 9 am and 9 pm

For peer review only

			• Mostly mental activity (such as reading, using a computer or phone, working or studying)<br>• Socialising or talking with other people<br><br>**2. How effortful was that activity?**<br><br>Sliding scale: 0 no effort to 10 most effortful	
**5.Psychological factors**	Stress<br>Repetitive thinking	Not at all/a Little/Moderately, Very.	**1. Thinking about the last hour, to what extent have you?**<br><br>• Felt stressed<br>• Worried about your illness in the future<br>• Thought about your illness in the past.	Delivered 6 times randomly stratified between 10am and 8pm.<br>Then at 9 am and pm
**6.Impact on daily functioning**	Severity and frequency?	Yes/no	**1. Thinking about the last hour have you experienced any of the following**<br><br>• Problems with personal care (washing/dressing)<br>• Problems doing usual activities<br>• Feeling exhausted after an activity	Delivered 6 times randomly stratified between 10am and 8pm.<br>Then at 9 am and pm

For peer review only

			• Problems walking about<br>• Problems communicating with others<br>• Problems concentrating or remembering things	
**7.Symptoms**	Frequency and severity		**1.** At the moment are you experiencing:<br><br>**Breathlessness** Yes/ no<br>If yes, it branches to the question: please rate how severe your breathlessness is:<br>***1 = Mild problem;***<br>***2 = Moderate problem;***<br>***3 = Severe problem;***<br><br>**2. Fatigue** yes/no<br>Branch as above.<br><br>**3. Pain/discomfort** yes/no<br>Branch as above.<br><br>**4. Dizziness or palpitations** yes/no<br>Branch as above.<br><br>**5. Feeling anxious** yes/no<br>Branch as above.<br><br>**6. Feeling depressed** yes/no<br>Branch as above.	Delivered 6 times randomly stratified between 10am and 8pm.<br>Then at 9 am and pm

For peer review only

**7.Change**	Improvement or decline in health status	Modified Patients' Global Impression of Change (PGIC)	See full assessment	onto redcap questionnai re
**8.Participatio n and activity**	Participation	Oxford Participation and Activities Questionnaire (OxPAQ)	See full assessment	onto redcap questionnai re
**9.Symptom burden and functional impact**	Multi dimensional	C19YRS	See full assessment	onto redcap questionnai re

Appendix 1: Ecological Momentary Assessment Ecological Momentary Assessment EMA assessment

For peer review only
